# Metastatic Disease to Clivus: Biopsy or Not?

**DOI:** 10.7759/cureus.5658

**Published:** 2019-09-14

**Authors:** Alessandra Cathel, Yasir R Khan, Danny Blais, Bandana Mahato, Deependra Mahato

**Affiliations:** 1 Neurosurgery, Desert Regional Medical Center, Palm Springs, USA

**Keywords:** hepatic tumor, clivus, metastatic cancer

## Abstract

Due to the aggressive nature of hepatocellular carcinoma (HCC), most patients succumb to disease before any distant metastasis, such as to the central nervous system (CNS), can occur. Thus only a handful of cases of metastasis to the skull base have been described. After a thorough review of the available literature published since 1950, we report the sixth case of HCC metastasis to the clivus. In this case, a 65-year-old man with a history of melanoma presented with sudden onset of right-sided headache and complete ophthalmoplegia of the right eye for one month. MRI of the brain with and without contrast demonstrated a homogeneously enhancing lesion involving the clivus with evidence of invasion into the right cavernous sinus. Through further body imaging, he was found to have an infiltrative lesion in the left hepatic lobe and underwent an ultrasound-guided biopsy of said lesion that was proven to be well-differentiated hepatocellular carcinoma. An endonasal endoscopic biopsy of his clival lesion was performed and the final pathology was consistent with a metastatic HCC. This case demonstrates the impact of obtaining a surgical specimen of clival tumors to confirm the suspected diagnosis, as well as to perform molecular studies that can drive post-operative decision-making and prognosis. As in this case, the final diagnosis altered treatment plans from that of melanoma, with systemic chemotherapy and radiosurgery, to stereotactic radiosurgery and intrahepatic radioembolization.

## Introduction

Tumors of the clivus are extremely rare, representing only 0.1-0.4% of all intracranial tumors, with chordomas and chondrosarcomas being the most frequent tumors of this region [1]. A small subset, only 56 cases reported in the literature, of clival tumors are metastatic lesions [2]. Hepatocellular carcinoma (HCC) is an extremely aggressive tumor associated with baseline poor endocrine and exocrine liver function, where most patients succumb to the disease within a few months of diagnosis. Due to its rapid course, distant metastases to the central nervous system (CNS) are extremely rare. The incidence of HCC metastasis to brain ranges from 0.26 to 2.2% but only a few cases of skull base metastasis have been reported in the literature [3]. When searching PubMed and Google Scholar using the keywords "hepatocellular carcinoma" and "clival metastasis"*,* only five cases of hepatocellular carcinoma to clivus were produced [1, 4-7]. Recent advances in intraarterial chemotherapy, kinase inhibitors like sorafenib, supportive care, and liver transplantation have led to increased overall survival for patients with HCC. Since the life expectancy of these patients is being extended, there is a potential for an increase in the rate of patients with intracranial involvement from HCC.

## Case presentation

**Figure 1:**
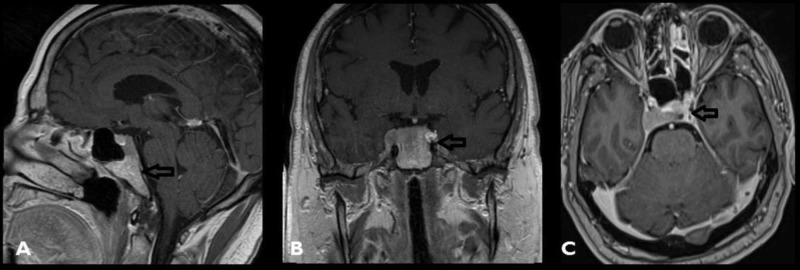
Different sections of an MRI T1 with a homogenous enhancing lesion involving the clivus (A-C); tumor invading right cavernous sinus (B, C)

A 65-year-old man with a history of melanoma had developed a sudden onset of right-sided headache and complete ophthalmoplegia of the right eye one month before he presented to our hospital. MRI of the brain with and without contrast was performed and demonstrated a homogenously enhancing lesion in the clivus with evidence of invasion into the right cavernous sinus (Figure 1). Initially, the clival lesion was thought to be a metastasis from melanoma, and he was planned to receive definitive radiation therapy. Prior to this treatment, additional metastatic work-up, including a CT of chest, abdomen, and pelvis with contrast, was conducted revealing an infiltrative lesion limited to the left hepatic lobe. Secondary to this finding, radiation was halted and he underwent an ultrasound-guided biopsy of the liver lesion, which was found to be well-differentiated HCC. Surprisingly, there was no previous medical history of cirrhosis and liver function tests were initially within normal limits. With two primary cancer diagnoses (melanoma and HCC) the decision was made to obtain a tissue diagnosis of the clival lesion. An endonasal endoscopic biopsy of his clival lesion was performed.

Operative Details

**Figure 2:**
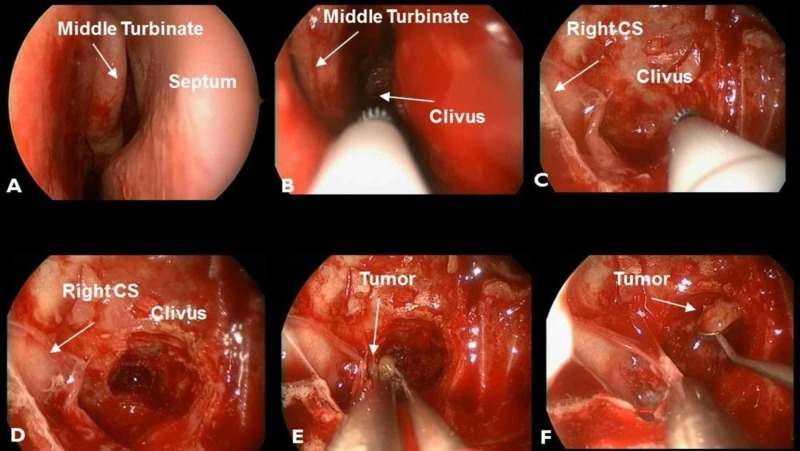
Endonasal endoscopic approach to the clivus A, B - initial inspection of right nasal cavity showing middle turbinate, septum and clivus. C, D - once the clivus and right cavernous sinus (CS) is localized, the clivus is drilled. E, F - using ring curette the tumor is removed and the final pathology was confirmed to be a hepatocellular carcinoma (HCC).

After administration of general anesthesia, the patient was placed in the supine position and the head was immobilized in a 3-pin Mayfield® holder (Integra LifeSciences Corporation, Plainsboro, NJ). A rigid zero degree endoscope was introduced into the right nostril and lidocaine 1% with epinephrine 1:100 was infiltrated into middle turbinate, inferior turbinate and septal mucosa for hemostasis. Using Penfield #1, the middle turbinate and inferior turbinate were lateralized. The sphenoid ostium was identified and the opening into the sphenoid sinus was enlarged. Anatomic landmarks within the sphenoid sinus were identified and these were confirmed with navigation. Using Cavitron® (Dentsply Sirona Canada, Woodbridge, Canada), the clivus was drilled adjacent to the right cavernous sinus. Beginning from the core of the lesion, the tumor was careful removed using a combination of pituitary forceps, curettes, and suction. Ring curettes were used to deliver tumor within the cavernous sinus (Figure 2). After adequate tumor samples were collected, copious amounts of irrigation were used, and hemostasis was obtained using a combination of Gelfoam powder and Gelfoam® (Pfizer, NYC). The surgical cavity was inspected to confirm that there was no cerebral spinal fluid (CSF) leak and to ensure the bleeding from cavernous sinus was stopped. An additional layer of fibrin glue and Gelfoam® was used to reinforce hemostasis and closure.

Pathology Result

**Figure 3:**
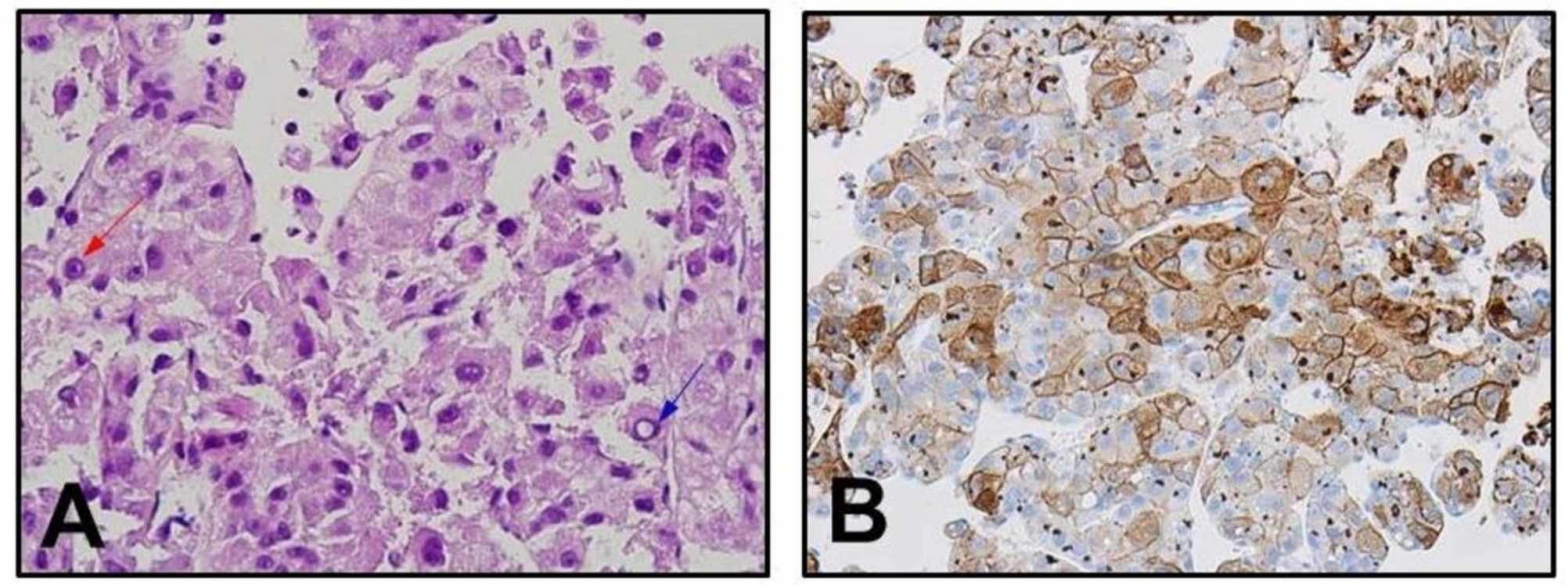
Pathology results A - polygonal tumor cells that resemble hepatocytes but with enlarged nuclei (high N/C ration) with prominent nucleoli (red arrow). Bile canaliculus is also seen here (blue arrow) with a distinct cell membrane. B - hepatocellular carcinoma (HCC) are positive for cytokeratin.

The cytomorphology of the specimen was consistent with the diagnosis of metastatic hepatocellular carcinoma. The hematoxylin and eosin (H&E) stain showed a polygonal tumor that resembled hepatocytes but with enlarged nuclei (high N/C ratio) and prominent nucleoli (Figure 3A). Immunohistochemistry further supported the diagnosis of HCC as the neoplastic cells were strongly positive for cytokeratin pan antibody (AE1/AE3) and heparan 1 (Figure 3B). Melanoma was excluded as a diagnosis by H&E stain and negative immunohistochemistry to melan-A.

## Discussion

Clival tumors represent 0.1-0.4% of all intracranial tumors, with chordomas and chondrosarcoma the most frequent tumors of this region [1]. The differential diagnosis of a clival tumor include chordoma, chondrosarcoma, lymphoma, plasmocytomas, metastatic lesions, meningioma, pituitary adenoma and nasopharyngeal carcinoma, as well as reconversion from yellow to red bone marrow [8]. Local tumor growth and the close proximity of surrounding critical structures, including the basilar artery, internal carotid artery, brain stem structures, and the cranial nerves, create a considerable morbidity and mortality burden if not timely treated. Even the most advanced neuroimaging techniques are not able to definitively differentiate between chordomatous and nonchordomatous lesions, with implications in terms of treatment decisions [9]. Isolated metastatic lesions of the clivus are an extremely rare subset of clival lesions, with only 56 cases reported in the literature. In these instances, the most common primary tumors are that of prostatic and renal carcinomas [2]. PubMed and Google Scholar search resulted in only five cases of clival metastasis arising from HCC, with this case being the sixth case reported in the literature [1, 4-7] (Table 1).

**Table 1:** Previously reported hepatocellular carcinoma cases available in the literature The present case has been added to this table. HCC - heptatocellular carcinoma; NA - not available; M - male; F - female; CN - cranial nerves

Authors & year	Age	Sex	Race	Symptoms	Primary tumor	Metastasis	Surgery	Adjuvant therapy
Sim RS, Tan HK (1994) [4]	40	M	Chinese	Postnasal drip with brownish sputum	HCC	Neg	Transsphenoidal biopsy	Radiations
Kim M, Na DL, Park SH, Jeon BS, Roh JK (1998) [5]	43	M	Korean	Left VI palsy	HCC	Lung	Transsphenoidal biopsy	Chemotherapy and radiation
Kim SR, Kanda F, Kobessho H, Sugimoto K, Matsuoka T, Kudo M, Hayashi Y (2006) [6]	50	F	Asian	Left VI palsy, right III and IV nerve palsy	HCC	Lungs	No biopsy	NA
Pallini R, Sabatino G, Doglietto F, Lauretti L, Fernandez E, Maira G (2009) [1]	69	M	NA	Right VI palsy and Left facial pain	HCC	NA	Transsphenoidal biopsy	NA
Nozaki I, Tsukada T, Nakamura Y, Takanaka T, Yamada M (2010) [7]	65	M	Japanese	Right VI, IX, X, and XII palsy	HCC	NA	No biopsy	Radiations
Present case, (2017)	65	M	White	Right CN III, IV, VI palsy	HCC, melanoma	Neg	Transsphenoidal biopsy	Radiations

Given that cranial nerve (CN) VI runs through Dorello’s canal in the clivus, it is not surprising that four out of five cases presented with CN VI palsy. Given that other CNs also run through the cavernous sinus, CN palsy should indicate extension of tumor into the cavernous sinus. Additionally, four of five previously described patients were of Asian descent, whereas this case presented that of a Caucasian [1, 4-7]. 

It is remarkable that in two out of these five cases, the clival mass was presumed to be HCC metastasis based on radiographic appearance alone, without obtaining a definitive pathological tissue diagnosis. However, based on our experience in this case, we firmly believe that a pathologic confirmation must be performed in any suspected metastatic clival mass, especially in those cases where two distinct primaries [10]. In addition, there may be instances where obtaining a biopsy of said lesion reveals a secondary primary malignancy not yet identified. 

Endoscopic endonasal approach for biopsy of lesions of the clivus carries a relatively low complication and mortality rate. In a total of 30 patients who underwent clival biopsies, there were no reports of cerebrospinal fluid (CSF) leak, meningitis, or encephalocele [11]. Furthermore, benefits of an endoscopic approach, as opposed to conventional open anterior or lateral routes, include faster recovery, shorter hospital stay, and minimal post-operative discomfort. Most importantly, it allows obtaining a tissue diagnosis that can dictate the course of adjuvant therapy without increased risk of intracranial tumor cell seeding, as the dura is not breached during this technique [12].

Hepatocellular carcinoma is the third leading cause of cancer mortality worldwide and its incidence has tripled in the United States from 1975 through 2005, with a marked increased incidence among middle-aged African-American, Hispanic, and Caucasian males. Although metastases from HCC are not uncommon, they mostly involve lungs (34-70%), lymph nodes (16-45%), bone (6%), and adrenal gland [3]. HCC metastases to CNS are rare, with a vertebral body and spinal epidural space being the most frequently affected anatomic locations [13]. The incidence of HCC metastases to the brain ranges from 0.26 to 2.2%, and the skull base the most rarely affected anatomic site in this regard [3, 5].

In terms of radiation therapy, pathologic disease alters treatment decisions. To manage brain metastases from HCCs, whole-brain radiotherapy (WBRT) alone or in addition to surgical resection has been used as standard treatments. Recently, due to the concerns of neurocognitive toxicity from WBRT, different series’ have proven that fractionated or single-dose stereotactic radiosurgery is effective in offering palliation and local control in these lesions affecting the brain or skull base and cranial nerves [14]. On the other hand, the poor response to conventional fractionated radiation of tumors such as melanoma which are radioresistant has led to several studies showing favorable rates of local control when high-dose stereotactic radiosurgery (SRS) is used as a treatment in selected patients with brain metastasis from melanoma [15]. Alternatively, for chordomas and chondrosarcomas high, focal, conventionally fractionated radiation doses as adjuvant therapy to extensive surgical resection is recommended [16].

Several prognostic systems and factors for prediction of overall survival in patients undergoing radiosurgery for brain metastases have proven to vary based on primary tumor site [17]. Consequently, its implication in clinical decision-making and stratification into clinical trials rely on the pathological diagnosis [18]. The diagnosis-specific Graded Prognostic Assessment score (ds-GPA), is a disease-specific prognostic classification that, although affected by selection bias and may not fully reflect outcomes incorporating modern systemic therapies, has undergone some independent validation and can identify patients with the worst prognosis best managed with palliative intent.

Systemic therapy decision making is drastically altered by tumor histology. For instance, systemic chemotherapy is not usually prescribed in advanced HCC (Stage C of Barcelona Clinic Liver Cancer [BCLC] system) due to its relative resistance, arising from a high rate expression of drug-resistance genes [18]. Also, because of the limited survival benefit and toxicity, systemic chemotherapy has a limited role in patients with hepatic dysfunction. An oral, small-molecule tyrosine kinase inhibitor, Sorafenib, has proven to increase the overall survival when used in patients with advanced HCC; however, as an antiangiogenic drug, its role in brain metastases has been limited due to increased risk of CNS hemorrhage.

Similar to other tumors harboring targetable genetic mutations such as non-small cells lung cancer (NSCLC) and renal cell carcinoma (RCC), and opposed to the systemic management of advanced HCC, an important role for immunotherapy and B-Raf and v-Raf murine sarcoma viral oncogene homolog B (BRAF) inhibitors as adjuvant to radiotherapy for brain metastases from melanoma has been reported in the literature [19, 20]. Treatments with high-dose interleukin-2 (IL-2), ipilimumab (monoclonal antibody anti-Cytotoxic T lymphocyte-associated antigen-4; cytotoxic T-lymphocyte-associated protein 4) and pembrolizumab and nivolumab (monoclonal antibodies anti-programmed cell death 1 protein) have been effective in patients with disseminated systemic melanoma and are also recommended for metastases involving the CNS [20]. However, it is worth highlighting that recent studies comparing molecular markers of matched primary tumors and brain metastases have shown significant differences between the two site’s molecular profiles, which further supports the importance of the histopathological and molecular characterization of the metastatic brain tumor cells [19].

Clival biopsy allows clinicians to confirm the histopathological diagnosis that can dictate the patient’s adjuvant therapy. The patient described in this report had a history of melanoma, but CT demonstrated a hepatic lesion, leading to the endoscopic endonasal biopsy. This then determined that the clival mass was a metastatic HCC, and only stereotactic radiotherapy (2000 cGy; five fractions) to the right posterior clival tumor and intrahepatic radioembolization to the left hepatic lobe with yttrium 90 radioisotope was performed. 

## Conclusions

Given the differential diagnosis array of clival lesions, and that most advanced neuroimaging techniques are not able to warrant an accurate and specific final diagnosis, endonasal endoscopic approach represents a safe and feasible option to obtain a mandatory pathological tissue diagnosis. This firm diagnosis will definitively impact the prognosis and further clinical decision-making in the setting of either metastatic disease, or newly diagnosed patients.
